# Supplementary material for: Targeted MicroRNA Profiling Reveals That Exendin-4 Modulates the Expression of Several MicroRNAs to Reduce Steatosis in HepG2 Cells
Source: Int J Mol Sci. 2023 Jul 18;24(14):11606. doi: 10.3390/ijms241411606 (PMC10380891; doi:10.3390/ijms241411606)
Supplement: Supplementary file 1 [file ijms-24-11606-s001.zip › ijms-2471972-supplementary.pdf]

Supplementary Materials

Targeted microRNA profiling reveals that Exendin-4 modulates the expression of several microRNAs to reduce steatosis in HepG2 cells

Olfa Khalifa[1], Khalid Ouararhni[2], Khaoula Errafii[3], Nehad M. Alajez[4,5], Abdelilah Arredouani [1,5]

[1]Diabetes Research Center, Qatar Biomedical Research Institute, Hamad Bin Khalifa University, Qatar Foundation, Doha, Qatar.

[2]Genomic core, Qatar Biomedical Research Institute, Hamad Bin Khalifa University, Qatar Foundation, Doha, Qatar.

[3]African Genome Center, Mohammed VI Polytechnic University (UM6P), Ben Guerir 43151, Morocco.

[4]Translational Cancer and Immunity Center Qatar Biomedical Research Institute, Hamad Bin Khalifa University, Qatar Foundation, Doha, Qatar.

[5]College of Health & Life Sciences, Hamad Bin Khalifa University Qatar Foundation, Doha, Qatar

*Corresponding author: aarredouani@hbku.edu.qa
Qatar Biomedical Research Institute
Hamad Bin Khalifa University
PO Box: 34110
Tel: +974 445 42947
Fax: +974 445 41770
Doha, Qatar

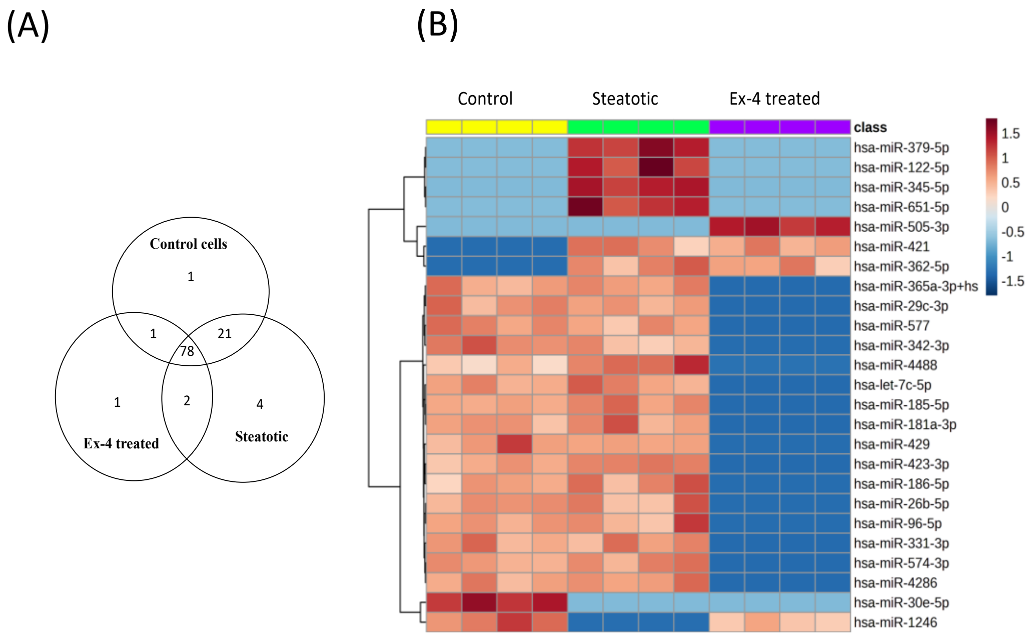

Supplementary Data S1. Heatmap and Venn diagram depicting microRNA expression. A. Venn diagram of the overlap of miRNA profiles in control, steatotic, and Ex-4-treated cells. The miRNAs differentially expressed in three are depicted in three overlapping circles. B. Heat map of the top 30 differentially expressed microRNAs between control, steatotic, and Ex-4-treated cells (n=4 for each condition). The miRNA species are shown on the right. Cluster analysis classified the samples into groups based on miRNA expression levels in each sample. The dendrogram shows significantly different expression levels of miRNAs among samples. Red indicates high miRNA expression, and bleu indicates relatively low miRNA expression.

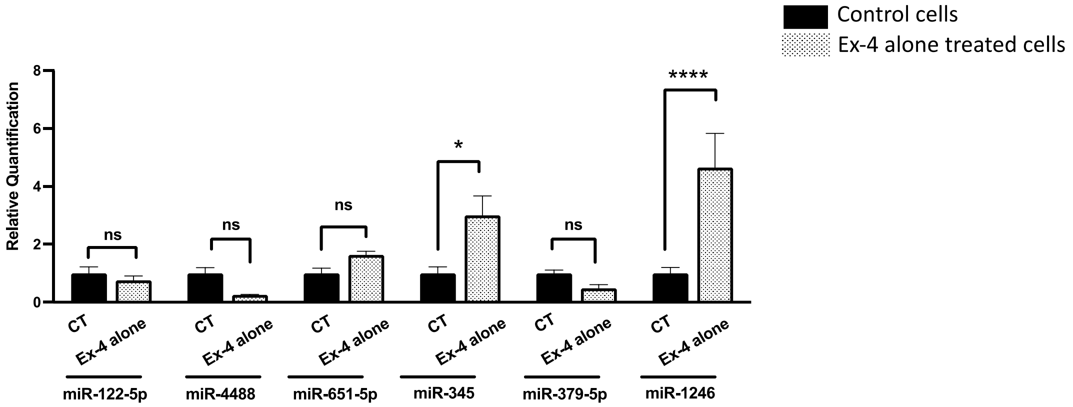

Supplementary dataS2: Ex-4 treatment downregulates the expression of miR-122-5p, miR-4488, and miR-379-5p and upregulates the expression of miR-651-5p, miR-345-5p, and miR-1246. mRNA expression levels of miR-122-5p, miR-4488, and miR-651-5p, miR-345, miR-379-5p and miR-1246. All values are expressed as the mean ± SE (n = 3). Ns: not significant, * $p < 0.05$, ** $p < 0.01$, *** $p < 0.001$. The experiment was performed in triplicate.

Table S1. List of miRNAs in CT, OA and Ex-4 group after normalization		
CT	OA	Ex-4
hsa-miR-769-5p	hsa-miR-122-5p	hsa-miR-769-5p
hsa-miR-197-3p	hsa-miR-345-5p	hsa-miR-30d-5p
hsa-miR-577	hsa-miR-769-5p	hsa-miR-505-3p
hsa-miR-145-5p	hsa-miR-197-3p	hsa-miR-302d-3p
hsa-miR-186-5p	hsa-miR-577	hsa-miR-362-5p
hsa-miR-30d-5p	hsa-miR-145-5p	hsa-miR-1246
hsa-miR-4488	hsa-miR-186-5p	hsa-miR-30e-3p
hsa-miR-429	hsa-miR-30d-5p	hsa-miR-95-3p
hsa-miR-302d-3p	hsa-miR-4488	hsa-miR-149-5p
hsa-miR-140-5p	hsa-miR-429	hsa-miR-574-5p
hsa-miR-10a-5p	hsa-miR-651-5p	hsa-miR-4532
hsa-miR-365a-3p+hsa-miR-365b-3p	hsa-miR-302d-3p	hsa-miR-374b-5p
hsa-miR-342-3p	hsa-miR-140-5p	hsa-miR-421
hsa-miR-29c-3p	hsa-miR-10a-5p	hsa-miR-590-5p
hsa-miR-1246	hsa-miR-365a-3p+hsa-miR-365b-3p	hsa-miR-30b-5p
hsa-miR-30e-3p	hsa-miR-342-3p	hsa-miR-92a-3p
hsa-miR-423-3p	hsa-miR-29c-3p	hsa-miR-34a-5p
hsa-miR-95-3p	hsa-miR-362-5p	hsa-miR-26a-5p
hsa-miR-149-5p	hsa-miR-30e-3p	hsa-miR-642a-5p
hsa-miR-574-3p	hsa-miR-423-3p	hsa-miR-132-3p
hsa-miR-30e-5p	hsa-miR-95-3p	hsa-miR-183-5p
hsa-miR-574-5p	hsa-miR-379-5p	hsa-miR-324-5p
hsa-miR-4532	hsa-miR-149-5p	hsa-miR-340-5p
hsa-let-7c-5p	hsa-miR-574-3p	hsa-let-7f-5p
hsa-miR-374b-5p	hsa-miR-574-5p	hsa-miR-142-3p
hsa-miR-1260a	hsa-miR-4532	hsa-miR-361-5p
hsa-miR-590-5p	hsa-let-7c-5p	hsa-miR-20a-5p+hsa-miR-20b-5p
hsa-miR-96-5p	hsa-miR-374b-5p	hsa-miR-31-5p
hsa-miR-4286	hsa-miR-1260a	hsa-miR-423-5p
hsa-miR-30b-5p	hsa-miR-421	hsa-miR-196a-5p
hsa-miR-92a-3p	hsa-miR-590-5p	hsa-miR-196b-5p
hsa-miR-34a-5p	hsa-miR-96-5p	hsa-miR-126-3p
hsa-miR-181a-3p	hsa-miR-4286	hsa-miR-301a-3p
hsa-miR-185-5p	hsa-miR-30b-5p	hsa-miR-151a-3p
hsa-miR-26a-5p	hsa-miR-92a-3p	hsa-miR-16-5p
hsa-miR-642a-5p	hsa-miR-34a-5p	hsa-miR-148b-3p
hsa-miR-132-3p	hsa-miR-181a-3p	hsa-miR-148a-3p
hsa-miR-183-5p	hsa-miR-185-5p	hsa-miR-18a-5p
hsa-miR-324-5p	hsa-miR-26a-5p	hsa-miR-28-5p
hsa-miR-331-3p	hsa-miR-642a-5p	hsa-miR-24-3p
hsa-miR-340-5p	hsa-miR-132-3p	hsa-miR-19b-3p
hsa-let-7f-5p	hsa-miR-183-5p	hsa-miR-107

hsa-miR-142-3p	hsa-miR-324-5p	hsa-miR-200a-3p
hsa-miR-361-5p	hsa-miR-331-3p	hsa-miR-106b-5p
hsa-miR-20a-5p+hsa-miR-20b-5p	hsa-miR-340-5p	hsa-miR-141-3p
hsa-miR-31-5p	hsa-let-7f-5p	hsa-miR-222-3p
hsa-miR-26b-5p	hsa-miR-142-3p	hsa-let-7e-5p
hsa-miR-423-5p	hsa-miR-361-5p	hsa-miR-98-5p
hsa-miR-196a-5p	hsa-miR-20a-5p+hsa-miR-20b-5p	hsa-miR-1180-3p
hsa-miR-196b-5p	hsa-miR-31-5p	hsa-miR-130a-3p
hsa-miR-126-3p	hsa-miR-26b-5p	hsa-miR-99b-5p
hsa-miR-301a-3p	hsa-miR-423-5p	hsa-miR-181a-5p
hsa-miR-151a-3p	hsa-miR-196a-5p	hsa-miR-23b-3p
hsa-miR-16-5p	hsa-miR-196b-5p	hsa-miR-27b-3p
hsa-miR-148b-3p	hsa-miR-126-3p	hsa-miR-19a-3p
hsa-miR-148a-3p	hsa-miR-301a-3p	hsa-miR-15a-5p
hsa-miR-18a-5p	hsa-miR-151a-3p	hsa-miR-221-3p
hsa-miR-28-5p	hsa-miR-16-5p	hsa-let-7i-5p
hsa-miR-24-3p	hsa-miR-148b-3p	hsa-miR-29a-3p
hsa-miR-19b-3p	hsa-miR-148a-3p	hsa-miR-9-5p
hsa-miR-107	hsa-miR-18a-5p	hsa-miR-125a-5p
hsa-miR-200a-3p	hsa-miR-28-5p	hsa-miR-106a-5p+hsa-miR-17-5p
hsa-miR-106b-5p	hsa-miR-24-3p	hsa-miR-32-5p
hsa-miR-141-3p	hsa-miR-19b-3p	hsa-let-7g-5p
hsa-miR-222-3p	hsa-miR-107	hsa-miR-200b-3p
hsa-let-7e-5p	hsa-miR-200a-3p	hsa-miR-7-5p
hsa-miR-98-5p	hsa-miR-106b-5p	hsa-miR-135b-5p
hsa-miR-1180-3p	hsa-miR-141-3p	hsa-let-7d-5p
hsa-miR-130a-3p	hsa-miR-222-3p	hsa-miR-15b-5p
hsa-miR-99b-5p	hsa-let-7e-5p	hsa-miR-25-3p
hsa-miR-181a-5p	hsa-miR-98-5p	hsa-miR-374a-5p
hsa-miR-23b-3p	hsa-miR-1180-3p	hsa-miR-125b-5p
hsa-miR-27b-3p	hsa-miR-130a-3p	hsa-miR-200c-3p
hsa-miR-19a-3p	hsa-miR-99b-5p	hsa-miR-23a-3p
hsa-miR-15a-5p	hsa-miR-181a-5p	hsa-let-7b-5p
hsa-miR-221-3p	hsa-miR-23b-3p	hsa-miR-93-5p
hsa-let-7i-5p	hsa-miR-27b-3p	hsa-miR-191-5p
hsa-miR-29a-3p	hsa-miR-19a-3p	hsa-miR-21-5p
hsa-miR-9-5p	hsa-miR-15a-5p	hsa-miR-100-5p
hsa-miR-125a-5p	hsa-miR-221-3p	hsa-miR-4454+hsa-miR-7975
hsa-miR-106a-5p+hsa-miR-17-5p	hsa-let-7i-5p	hsa-let-7a-5p
hsa-miR-32-5p	hsa-miR-29a-3p	hsa-miR-29b-3p
hsa-let-7g-5p	hsa-miR-9-5p	
hsa-miR-200b-3p	hsa-miR-125a-5p	
hsa-miR-7-5p	hsa-miR-106a-5p+hsa-miR-17-5p	
hsa-miR-135b-5p	hsa-miR-32-5p	

hsa-let-7d-5p	hsa-let-7g-5p	
hsa-miR-15b-5p	hsa-miR-200b-3p	
hsa-miR-25-3p	hsa-miR-7-5p	
hsa-miR-374a-5p	hsa-miR-135b-5p	
hsa-miR-125b-5p	hsa-let-7d-5p	
hsa-miR-200c-3p	hsa-miR-15b-5p	
hsa-miR-23a-3p	hsa-miR-25-3p	
hsa-let-7b-5p	hsa-miR-374a-5p	
hsa-miR-93-5p	hsa-miR-125b-5p	
hsa-miR-191-5p	hsa-miR-200c-3p	
hsa-miR-21-5p	hsa-miR-23a-3p	
hsa-miR-100-5p	hsa-let-7b-5p	
hsa-miR-4454+hsa-miR-7975	hsa-miR-93-5p	
hsa-let-7a-5p	hsa-miR-191-5p	
hsa-miR-29b-3p	hsa-miR-21-5p	
	hsa-miR-100-5p	
	hsa-miR-4454+hsa-miR-7975	
	hsa-let-7a-5p	
	hsa-miR-29b-3p	

Table S2. miRNAs with VIP score > 1 from PLS-DA analysis of OA versus control samples.				
miRNAs	Comp. 1	Comp. 2	Comp. 3	Comp. 4
hsa-miR-345-5p	1.8584	1.7905	1.7882	1.788
hsa-miR-30e-5p	1.8555	1.7915	1.7891	1.789
hsa-miR-1246	1.8509	1.7839	1.7816	1.7814
hsa-miR-379-5p	1.8492	1.7835	1.7811	1.781
hsa-miR-362-5p	1.8409	1.7711	1.7687	1.7686
hsa-miR-651-5p	1.8351	1.7677	1.7654	1.7653
hsa-miR-421	1.8349	1.7792	1.7769	1.7768
hsa-miR-122-5p	1.8249	1.7629	1.7606	1.7604
hsa-miR-4532	1.7495	1.6814	1.6791	1.679
hsa-let-7a-5p	1.663	1.5993	1.5971	1.597
hsa-miR-4488	1.6577	1.5932	1.5914	1.5913
hsa-miR-191-5p	1.6038	1.5448	1.5428	1.5427
hsa-let-7b-5p	1.5708	1.5164	1.5144	1.5143
hsa-miR-29b-3p	1.5582	1.5025	1.5006	1.5005
hsa-miR-423-3p	1.5429	1.4954	1.4947	1.4946
hsa-miR-200a-3p	1.5357	1.4774	1.4756	1.4755
hsa-miR-374a-5p	1.53	1.4708	1.4703	1.4702
hsa-miR-423-5p	1.4925	1.4385	1.4366	1.4365
hsa-let-7e-5p	1.4786	1.421	1.4193	1.4192
hsa-miR-302d-3p	1.4609	1.4061	1.4055	1.4054
hsa-miR-342-3p	1.388	1.3624	1.3613	1.3612
hsa-miR-98-5p	1.383	1.3302	1.3285	1.3284
hsa-miR-185-5p	1.3716	1.3299	1.3295	1.3294
hsa-miR-23a-3p	1.3199	1.279	1.2777	1.2777
hsa-miR-7-5p	1.3187	1.2736	1.2719	1.2719
hsa-miR-95-3p	1.2725	1.2324	1.2307	1.2307
hsa-miR-148a-3p	1.1975	1.1699	1.1686	1.1685
hsa-miR-135b-5p	1.1948	1.1529	1.1538	1.1538

hsa-miR-24-3p	1.1945	1.1579	1.1564	1.1563
hsa-miR-21-5p	1.183	1.1676	1.166	1.166
hsa-miR-149-5p	1.1341	1.1025	1.1025	1.1026
hsa-miR-25-3p	1.1108	1.0903	1.0889	1.0888
hsa-let-7g-5p	1.1037	1.0845	1.0853	1.0852
hsa-miR-126-3p	1.0964	1.0605	1.0596	1.0596
hsa-miR-577	1.0637	1.0472	1.0459	1.0458
hsa-miR-221-3p	1.0496	1.1099	1.1091	1.109
hsa-miR-26a-5p	1.0425	1.0173	1.0163	1.0162
hsa-miR-100-5p	1.0229	1.0402	1.0391	1.039
hsa-miR-30e-3p	1.0198	0.9801	0.98017	0.98037
hsa-miR-31-5p	1.0159	0.97639	0.97857	0.97861
hsa-miR-186-5p	1.0117	0.99637	0.99717	0.9971
hsa-miR-30b-5p	1.0114	0.98978	0.99066	0.99065

Table S3. miRNAs with VIP score > 1 from PLS-DA analysis of Ex-4 versus OA samples.				
miRNAs	Comp. 1	Comp. 2	Comp. 3	Comp. 4
hsa-miR-429	1.2876	1.2637	1.2616	1.2614
hsa-miR-423-3p	1.2874	1.2644	1.2623	1.2621
hsa-miR-29c-3p	1.2851	1.2586	1.2565	1.2564
hsa-miR-505-3p	1.2845	1.2689	1.2668	1.2667
hsa-miR-365a-3p+hsa-miR-365b-3p	1.2841	1.2513	1.2491	1.2489
hsa-miR-345-5p	1.2833	1.2525	1.2502	1.2501
hsa-miR-185-5p	1.2827	1.2586	1.2566	1.2565
hsa-miR-4286	1.2824	1.2473	1.2451	1.245
hsa-miR-1246	1.2816	1.2673	1.265	1.2649
hsa-miR-574-3p	1.2799	1.2467	1.2444	1.2443
hsa-miR-577	1.2787	1.2536	1.2513	1.2512
hsa-miR-4488	1.2783	1.2436	1.2417	1.2415
hsa-miR-379-5p	1.277	1.2548	1.2526	1.2525
hsa-miR-331-3p	1.2764	1.2538	1.2523	1.2522
hsa-miR-342-3p	1.275	1.2443	1.242	1.2419
hsa-let-7c-5p	1.2745	1.2579	1.2556	1.2555
hsa-miR-181a-3p	1.2728	1.2555	1.2537	1.2535
hsa-miR-197-3p	1.2715	1.2595	1.2582	1.2581
hsa-miR-186-5p	1.27	1.2303	1.228	1.2279
hsa-miR-651-5p	1.2672	1.2333	1.2312	1.2311
hsa-miR-1260a	1.2611	1.2421	1.24	1.2399
hsa-miR-122-5p	1.2602	1.248	1.2457	1.2456
hsa-miR-10a-5p	1.2593	1.2457	1.2441	1.2441
hsa-miR-26b-5p	1.2593	1.2146	1.2123	1.2122
hsa-miR-96-5p	1.2551	1.2092	1.207	1.2069
hsa-miR-140-5p	1.2539	1.2462	1.2456	1.2454
hsa-miR-145-5p	1.2482	1.2146	1.2129	1.2129
hsa-miR-423-5p	1.2466	1.2528	1.2505	1.2503

hsa-let-7i-5p	1.2297	1.1801	1.1782	1.1781
hsa-miR-4454+hsa-miR-7975	1.2043	1.1557	1.1543	1.1542
hsa-let-7g-5p	1.1791	1.132	1.1311	1.131
hsa-let-7a-5p	1.1718	1.1265	1.1247	1.1246
hsa-miR-1180-3p	1.1603	1.1134	1.1123	1.1122
hsa-miR-30b-5p	1.156	1.1104	1.1105	1.1104
hsa-let-7e-5p	1.147	1.1014	1.0995	1.0994
hsa-miR-21-5p	1.1456	1.1077	1.1058	1.1057
hsa-miR-4532	1.1401	1.0975	1.0963	1.0963
hsa-let-7b-5p	1.1355	1.1016	1.0999	1.0998
hsa-miR-340-5p	1.1348	1.1044	1.1026	1.1025
hsa-miR-181a-5p	1.107	1.063	1.061	1.0609
hsa-miR-106b-5p	1.1012	1.0569	1.0549	1.0549
hsa-miR-29b-3p	1.0991	1.0669	1.0653	1.0652
hsa-miR-200c-3p	1.0901	1.0494	1.048	1.0483
hsa-miR-200a-3p	1.0781	1.0439	1.0428	1.0427
hsa-miR-24-3p	1.0726	1.0338	1.0336	1.0339
hsa-miR-7-5p	1.0664	1.0233	1.0216	1.0216
hsa-miR-141-3p	1.0624	1.0422	1.0408	1.0407
hsa-miR-26a-5p	1.0617	1.0242	1.0223	1.0224
hsa-let-7f-5p	1.055	1.0197	1.0197	1.0196
hsa-miR-106a-5p+hsa-miR-17-5p	1.0435	1.02	1.0181	1.018
hsa-miR-25-3p	1.0414	1.017	1.0152	1.015
hsa-miR-99b-5p	1.0409	1.0195	1.0196	1.0195
hsa-miR-191-5p	1.0273	1.0063	1.0068	1.0067
hsa-miR-30e-3p	1.0222	1.0114	1.0127	1.0127
hsa-miR-29a-3p	1.0116	1.0095	1.0085	1.0085
hsa-miR-374a-5p	1.0055	0.9709	0.96912	0.96909
hsa-let-7d-5p	1.0045	1.0019	1.0003	1.0002

Table S4. NanoString Data.

sample	C1	C2	C3	C4	OA1	OA2	OA3	OA4	EX1	EX2	EX3	EX4
label	1	1	1	1	2	2	2	2	3	3	3	3
hsa-miR-122-5p					4.576651092	3.262642741	6.82716493	3.640145606				
hsa-miR-345-5p					4.250797024	3.262642741	3.95256917	4.160166407				
hsa-miR-769-5p	5.847953216	4.14507772	3.266194883	4.41974194	4.126268918	3.188097768	5.03054258	4.160166407	4.076513013	4.08496732	5.353319058	4.504933131
hsa-miR-197-3p	4.948268106	3.799654577	8.169934641	5.639285775	4.250797024	8.156606852	6.10851599	5.720228809				
hsa-miR-577	5.847953216	5.18134715	4.08496732	5.038089229	4.151117242	3.262642741	5.03054258	4.160166407				
hsa-miR-145-5p	4.498425551	4.490500864	4.08496732	6.532389766	6.866175182	9.564293305	3.23392023	7.800312012				
hsa-miR-186-5p	3.598740441	5.872193437	5.443658138	4.971530672	7.438894793	4.350190321	6.46784046	8.840353614				
hsa-miR-30d-5p	3.598740441	6.21761658	11.9760479	7.264134975	7.970244421	4.893964111	7.54581387	10.92043682	5.017246786	3.267973856	5.353319058	4.5461799
hsa-miR-505-3p									3.762935089	4.08496732	3.211991435	3.686631281
hsa-miR-4488	5.847953216	4.835924007	7.352941176	4.899292324	9.564293305	11.41924959	11.13905857	17.16068643				
hsa-miR-429	3.598740441	4.490500864	7.352941176	4.354926511	4.250797024	4.350190321	4.160166407	4.253717917				
hsa-miR-651-5p					7.438894793	3.806416531	4.67121811	5.200208008				
hsa-miR-302d-3p	4.048582996	5.872193437	4.899292324	4.940022919	7.221069685	5.437737901	7.90513834	8.320332813	4.703668862	6.535947712	6.423982869	5.887866481
hsa-miR-140-5p	3.148897886	3.799654577	3.267973856	3.405508773	3.188097768	7.612833061	3.95256917	4.160166407				
hsa-miR-10a-5p	4.048582996	4.14507772	6.535947712	5.988023952	3.719447396	5.981511691	8.98311175	6.24024961				

**hsa-miR-365a-3p+hsa-miR-365b-3p**	8.547008547	5.872193437	5.443658138	6.620953374	7.438894793	6.525285481	6.10851599	7.800312012				
**hsa-miR-342-3p**	6.887494493	8.547008547	6.12745098	5.988023952	6.376195537	4.350190321	3.95256917	4.680187207				
**hsa-miR-29c-3p**	6.297795771	3.799654577	4.901960784	5.443658138	4.508906829	4.893964111	3.95256917	4.680187207				
**hsa-miR-362-5p**					4.84447831	3.262642741	5.03054258	6.24024961	4.157509995	4.08496732	5.353319058	3.034243606
**hsa-miR-1246**	4.498425551	5.18134715	7.621121394	5.766964698					3.135779241	4.08496732	3.211991435	3.034243606
**hsa-miR-30e-3p**	3.148897886	4.835924007	3.266194883	3.750338925	4.782146652	3.806416531	5.74919152	4.160166407	3.762935089	3.267973856	3.211991435	3.034243606
**hsa-miR-423-3p**	3.148897886	3.799654577	4.493464052	3.814005505	4.782146652	4.893964111	5.200208008	4.958772924				
**hsa-miR-95-3p**	5.398110661	6.21761658	5.310457516	6.532389766	7.438894793	5.981511691	6.82716493	7.800312012	5.33082471	5.718954248	3.034243606	4.694674188
**hsa-miR-379-5p**					4.782146652	4.350190321	6.760270411	5.297535795				
**hsa-miR-149-5p**	5.847953216	7.944732297	5.310457516	8.165487207	5.056549415	6.376195537	3.5932447	5.200208008	3.762935089	4.901960784	8.565310493	3.467706979
**hsa-miR-574-3p**	4.048582996	3.799654577	3.267973856	3.266194883	4.060059539	3.188097768	4.31189364	4.680187207				
**hsa-miR-30e-5p**	3.598740441	4.835924007	3.676470588	4.354926511								
**hsa-miR-574-5p**	6.297795771	5.526770294	4.493464052	7.07675558	6.376195537	4.350190321	5.03054258	7.800312012	4.703668862	3.267973856	5.353319058	3.901170351
**hsa-miR-4532**	7.647323437	5.872193437	6.535947712	5.988023952	11.15834219	10.33170201	11.49838304	14.04056162	5.017246786	8.169934641	4.282655246	3.901170351
**hsa-let-7c-5p**	4.048582996	4.835924007	3.676470588	3.810560697	5.844845909	4.893964111	3.95256917	3.640145606				
**hsa-miR-374b-5p**	5.847953216	6.21761658	4.901960784	4.354926511	4.250797024	8.156606852	5.03054258	8.840353614	7.212292255	3.267973856	3.211991435	3.901170351

**hsa-miR-1260a**	24.29149798	8.635578584	6.944444444	6.532389766	4.782146652	4.893964111	9.360374415	6.345495059				
**hsa-miR-421**					5.844845909	5.981511691	5.03054258	3.120124805	4.076513013	5.718954248	3.901170351	4.565545871
**hsa-miR-590-5p**	6.747638327	7.599309154	5.718954248	4.899292324	3.719447396	5.981511691	3.23392023	7.280291212	5.407966831	6.535947712	5.353319058	4.334633723
**hsa-miR-96-5p**	4.948268106	5.526770294	4.493464052	5.443658138	5.714371035	4.350190321	3.95256917	8.840353614				
**hsa-miR-4286**	3.598740441	4.835924007	3.266194883	3.900286444	4.250797024	3.806416531	3.95256917	5.200208008				
**hsa-miR-30b-5p**	7.197480882	7.25388601	8.578431373	5.988023952	7.438894793	8.700380642	7.90513834	12.48049922	4.703668862	4.901960784	5.353319058	4.768097096
**hsa-miR-92a-3p**	7.197480882	8.29015544	9.395424837	7.621121394	7.438894793	7.612833061	6.46784046	14.56058242	7.212292255	4.08496732	4.768097096	5.35511889
**hsa-miR-34a-5p**	8.547008547	5.526770294	3.676470588	5.443658138	9.032943677	5.437737901	5.38986705	9.880395216	4.703668862	4.901960784	4.282655246	5.201560468
**hsa-miR-181a-3p**	4.086028162	4.498425551	4.493464052	3.266194883	4.709868881	6.376195537	3.5932447	4.160166407				
**hsa-miR-185-5p**	3.898394198	3.799654577	4.08496732	3.810560697	4.690164759	5.437737901	3.95256917	4.680187207				
**hsa-miR-26a-5p**	15.29464687	8.981001727	7.352941176	8.165487207	10.62699256	10.33170201	17.60689903	22.88091524	8.153026027	4.901960784	7.494646681	5.63502384
**hsa-miR-642a-5p**	6.297795771	5.526770294	4.493464052	4.354926511	6.16033923	4.250797024	5.38986705	8.840353614	6.898714331	6.535947712	6.423982869	5.63502384
**hsa-miR-132-3p**	5.370589993	4.948268106	7.352941176	3.810560697	4.897168193	4.782146652	5.74919152	4.160166407	4.769256675	4.390090938	4.282655246	5.63502384
**hsa-miR-183-5p**	8.097165992	8.635578584	11.4379085	8.709853021	5.313496281	5.981511691	7.90513834	13.00052002	4.703668862	6.535947712	9.635974304	6.068487213
**hsa-miR-324-5p**	4.948268106	6.908462867	5.310457516	5.988023952	6.907545165	3.806416531	5.03054258	8.320332813	4.390090938	3.267973856	7.494646681	6.068487213
**hsa-miR-331-3p**	6.09678374	7.944732297	4.901960784	5.443658138	4.782146652	7.612833061	5.74919152	6.760270411				
**hsa-miR-340-5p**	9.446693657	11.39896373	10.2124183	15.7866086	10.09564293	14.13811854	12.21703198	10.40041602	6.585136406	7.352941176	4.282655246	6.501950585

hsa-let-7f-5p	17.9937022	15.19861831	13.88888889	4.899292324	24.44208289	9.244154432	11.49838304	22.36089444	5.957980558	7.352941176	6.501950585	6.604290773
hsa-miR-142-3p	8.547008547	8.29015544	6.535947712	10.34295046	7.438894793	7.612833061	7.1864894	14.04056162	5.644402634	8.986928105	4.282655246	6.935413958
hsa-miR-361-5p	9.446693657	10.70811744	6.535947712	8.165487207	7.970244421	8.156606852	7.90513834	14.04056162	6.898714331	8.986928105	9.635974304	7.36887733
hsa-miR-20a-5p+hsa-miR-20b-5p	22.94197031	25.90673575	19.60784314	16.87534023	22.84803401	17.94453507	16.16960115	26.00104004	13.17027281	18.79084967	6.423982869	7.36887733
hsa-miR-31-5p	5.847953216	5.872193437	3.267973856	9.798584649	9.564293305	8.156606852	6.82716493	9.360374415	5.33082471	3.267973856	3.211991435	7.36887733
hsa-miR-26b-5p	5.847953216	7.599309154	7.352941176	7.621121394	8.501594049	5.437737901	5.38986705	10.40041602				
hsa-miR-423-5p	8.547008547	10.3626943	7.761437908	9.798584649	12.75239107	10.8754758	13.29500539	10.92043682	6.585136406	6.535947712	7.36887733	6.82998715
hsa-miR-196a-5p	11.69590643	12.08981002	7.761437908	8.709853021	11.15834219	13.05057096	13.29500539	9.880395216	10.03449357	13.07189542	7.494646681	9.102730819
hsa-miR-196b-5p	12.14574899	13.47150259	8.578431373	4.354926511	9.564293305	3.806416531	12.21703198	13.52054082	9.407337723	13.07189542	5.353319058	9.102730819
hsa-miR-126-3p	11.24606388	13.47150259	16.33986928	13.06477953	15.94048884	17.94453507	13.29500539	23.92095684	12.54311696	15.52287582	11.77730193	9.536194192
hsa-miR-301a-3p	17.09401709	17.61658031	17.56535948	15.24224279	20.72263549	14.68189233	17.60689903	24.96099844	12.85669489	18.79084967	8.565310493	9.536194192
hsa-miR-151a-3p	11.24606388	11.74438687	9.803921569	12.52041372	14.87778959	19.57585644	7.54581387	19.76079043	9.093759799	10.62091503	7.494646681	9.536194192
hsa-miR-16-5p	21.14260009	27.97927461	15.93137255	18.50843767	19.12858661	10.33170201	12.93568092	25.48101924	14.73816243	6.535947712	6.423982869	9.969657564
hsa-miR-148b-3p	17.54385965	12.08981002	15.11437908	11.43168209	12.22104145	11.96302338	15.81027668	20.80083203	12.85669489	13.88888889	12.84796574	10.83658431
hsa-miR-148a-3p	16.64417454	17.96200345	15.11437908	10.34295046	19.12858661	18.48830886	17.60689903	26.52106084	13.48385074	17.97385621	11.77730193	10.83658431

**hsa-miR-18a-5p**	16.19433198	23.83419689	17.97385621	21.23026674	17.53453773	14.13811854	17.24757456	21.84087363	15.67889621	20.4248366	10.70663812	10.83658431
**hsa-miR-28-5p**	17.54385965	14.85319516	13.48039216	14.15351116	17.53453773	13.05057096	16.88825009	13.52054082	13.48385074	10.62091503	12.84796574	11.27004768
**hsa-miR-24-3p**	10.34637877	15.88946459	11.4379085	10.88731628	19.12858661	14.13811854	12.93568092	17.16068643	10.97522734	9.803921569	11.77730193	11.27004768
**hsa-miR-19b-3p**	15.74448943	21.07081174	11.4379085	16.33097441	11.68969182	13.59434475	10.7797341	25.48101924	12.22953904	8.986928105	11.77730193	11.27004768
**hsa-miR-107**	20.24291498	19.34369603	16.74836601	15.7866086	21.78533475	17.40076128	11.13905857	29.64118565	13.79742866	14.70588235	6.423982869	11.27004768
**hsa-miR-200a-3p**	18.44354476	17.61658031	14.29738562	19.5971693	22.84803401	22.29472539	22.63744161	28.60114405	19.12825337	15.52287582	10.70663812	11.70351105
**hsa-miR-106b-5p**	19.79307242	25.90673575	10.62091503	16.33097441	19.65993624	24.46982055	17.60689903	21.32085283	15.36531828	13.88888889	9.635974304	12.13697443
**hsa-miR-141-3p**	26.99055331	32.46977547	26.55228758	27.76265651	28.16153029	28.82001088	28.7459576	40.04160166	19.12825337	24.50980392	13.91862955	13.43736454
**hsa-miR-222-3p**	20.24291498	17.61658031	17.97385621	10.88731628	22.84803401	18.48830886	15.45095221	19.76079043	14.11100659	11.4379085	16.05995717	13.87082792
**hsa-let-7e-5p**	16.19433198	20.37996546	15.93137255	21.77463255	28.16153029	22.29472539	23.35609055	27.04108164	8.780181875	16.33986928	9.635974304	13.87082792
**hsa-miR-98-5p**	22.49212776	21.41623489	20.83333333	28.30702232	30.2869288	26.10114192	28.38663313	35.36141446	18.1875196	28.59477124	17.13062099	14.73775466
**hsa-miR-1180-3p**	14.84480432	18.99827288	12.66339869	17.41970604	18.06588735	16.3132137	16.16960115	18.72074883	14.11100659	13.88888889	13.91862955	14.73775466
**hsa-miR-130a-3p**	19.79307242	23.83419689	22.05882353	13.06477953	15.94048884	23.92604676	22.99676608	28.60114405	13.17027281	17.97385621	6.423982869	15.17121803
**hsa-miR-99b-5p**	37.33693207	34.19689119	28.18627451	31.02885139	30.81827843	26.64491572	26.23068631	39.00156006	16.9332079	25.32679739	19.27194861	16.03814478
**hsa-miR-181a-5p**	35.0877193	27.28842832	18.79084967	33.75068046	41.44527099	26.64491572	37.72906935	45.76183047	20.38256507	23.69281046	23.55460385	17.77199827
**hsa-miR-23b-3p**	34.63787674	33.50604491	23.69281046	27.21829069	36.13177471	23.92604676	24.79338843	36.92147686	23.51834431	26.14379085	31.04925054	19.07238838

**hsa-miR-27b-3p**	30.13945119	33.85146805	25.73529412	26.12955906	29.22422954	25.01359434	24.07473949	24.96099844	20.06898714	18.79084967	7.494646681	19.50585176
**hsa-miR-19a-3p**	20.24291498	46.63212435	29.41176471	31.02885139	27.63018066	33.1702012	21.20014373	39.52158086	22.89118846	30.22875817	20.34261242	19.93931513
**hsa-miR-15a-5p**	23.84165542	32.12435233	20.4248366	25.58519325	29.75557917	25.55736813	24.07473949	26.52106084	18.50109752	28.59477124	20.34261242	24.27394885
**hsa-miR-221-3p**	62.97795771	57.68566494	38.80718954	52.80348394	38.78852285	36.43284394	29.10528207	56.68226729	32.92568203	41.66666667	32.11991435	25.1408756
**hsa-let-7i-5p**	58.92937472	51.46804836	42.48366013	47.3598258	52.07226355	53.83360522	47.79015451	67.08268331	26.34054563	30.22875817	22.48394004	25.57433897
**hsa-miR-29a-3p**	57.1300045	69.43005181	49.83660131	54.9809472	46.75876727	47.30831974	41.68163852	68.12272491	26.65412355	43.30065359	21.41327623	25.57433897
**hsa-miR-9-5p**	32.83850652	39.0328152	32.67973856	29.39575395	31.88097768	33.1702012	29.82393101	40.04160166	29.78990279	37.58169935	22.48394004	26.00780234
**hsa-miR-125a-5p**	46.33378318	50.08635579	43.30065359	48.44855743	49.41551541	46.76454595	53.53934603	73.84295372	38.25650674	40.8496732	17.13062099	27.74165583
**hsa-miR-106a-5p+hsa-miR-17-5p**	71.07512371	71.84801382	56.78104575	62.60206859	66.95005313	62.53398586	61.0851599	80.60322413	49.85888993	50.65359477	34.26124197	28.1751192
**hsa-miR-32-5p**	38.68645974	31.0880829	35.94771242	44.09363092	36.13177471	38.06416531	25.51203737	36.40145606	29.47632487	46.56862745	23.55460385	29.47550932
**hsa-let-7g-5p**	82.77103014	77.02936097	74.34640523	64.77953185	83.4218916	72.32191408	103.1261229	120.6448258	49.23173409	32.67973856	24.62526767	32.50975293
**hsa-miR-200b-3p**	42.28520018	48.01381693	42.89215686	24.49646162	45.69606801	28.27623709	45.27488322	48.88195528	35.74788335	32.67973856	28.90792291	32.9432163
**hsa-miR-7-5p**	53.0814215	41.4507772	44.52614379	41.91616766	61.63655685	45.67699837	59.28853755	70.72282891	40.13797429	39.21568627	46.0385439	37.27785002
**hsa-miR-135b-5p**	54.43094917	49.74093264	50.24509804	56.61404464	61.63655685	57.09624796	52.82069709	65.0026001	43.27375353	53.10457516	31.04925054	40.31209363
**hsa-let-7d-5p**	90.86819613	84.62867012	80.47385621	93.08655416	104.6758767	67.97172376	74.73948976	103.4841394	53.3082471	72.7124183	48.17987152	44.21326398
**hsa-miR-15b-5p**	69.27575349	86.70120898	69.85294118	82.19923789	78.63974495	64.16530723	56.05461732	78.52314093	47.97742239	59.64052288	35.33190578	44.21326398

**hsa-miR-25-3p**	94.46693657	103.626943	84.96732026	76.21121394	104.6758767	101.6856987	92.70571326	113.8845554	67.41925368	88.23529412	48.17987152	67.18682271
**hsa-miR-374a-5p**	89.06882591	98.10017271	77.20588235	86.00979858	115.3028693	119.6302338	97.73625584	109.2043682	83.72530574	99.67320261	64.23982869	80.19072388
**hsa-miR-125b-5p**	173.1893837	197.582038	148.6928105	174.7414262	177.4707758	136.4872213	120.7330219	197.0878835	108.8115397	142.9738562	111.3490364	94.49501517
**hsa-miR-200c-3p**	129.5546559	143.6960276	122.5490196	127.3816004	151.434644	136.4872213	112.4685591	139.8855954	107.8708059	107.0261438	92.07708779	94.49501517
**hsa-miR-23a-3p**	123.2568601	108.1174439	110.7026144	100.7076756	143.9957492	110.3860794	151.2756019	178.3671347	99.40420194	144.6078431	110.2783726	97.52925878
**hsa-let-7b-5p**	139.9010346	141.6234888	122.9575163	127.3816004	173.7513284	166.3947798	163.4926338	222.5689028	111.3201631	137.254902	96.35974304	98.82964889
**hsa-miR-93-5p**	127.3054431	150.9499136	114.7875817	143.7125749	153.5600425	135.3996737	116.0618038	175.2470099	106.3029163	131.5359477	73.875803	100.5635024
**hsa-miR-191-5p**	116.5092218	133.6787565	116.4215686	117.5830158	153.0286929	151.1691136	140.1365433	177.8471139	114.1423644	141.3398693	119.9143469	123.9705245
**hsa-miR-21-5p**	309.0418354	291.1917098	279.003268	269.4610778	370.3506908	278.9559543	329.500539	428.4971399	172.4678583	219.7712418	115.6316916	133.5067187
**hsa-miR-100-5p**	219.0733243	243.8687392	212.0098039	187.26184	256.1105207	217.509516	227.093065	303.6921477	190.6553779	236.1111111	164.882227	166.8833984
**hsa-miR-4454+hsa-miR-7975**	1262.708052	342.6597582	336.6013072	344.5835602	359.1923486	384.4480696	359.6837945	456.5782631	244.5907808	245.9150327	217.3447537	186.3892501
**hsa-let-7a-5p**	808.3670715	812.08981	736.1111111	688.6227545	977.151966	963.0233823	948.6166008	1136.24545	546.5663217	714.0522876	431.4775161	465.1061985
**hsa-miR-29b-3p**	805.6680162	890.5008636	747.9575163	749.0473598	1026.567481	930.9407287	911.9655049	1073.842954	693.9479461	776.9607843	474.3040685	578.2401387
